# Advancements in Research on Duck Tembusu Virus Infections

**DOI:** 10.3390/v16050811

**Published:** 2024-05-20

**Authors:** Yuting Cheng, Ruoheng Wang, Qingguo Wu, Jinying Chen, Anping Wang, Zhi Wu, Fang Sun, Shanyuan Zhu

**Affiliations:** 1 Engineering Technology Research Center for Modern Animal Science and Novel Veterinary Pharmaceutic Development, Jiangsu Key Laboratory of Veterinary Bio-Pharmaceutical High Technology Research, Jiangsu Agri-Animal Husbandry Vocational College, Taizhou 225300, China; yuting920428@whu.edu.cn (Y.C.);; 2 Department of Biochemistry and Molecular Biology, College of Basic Medicine, Hubei University of Medicine, Shiyan 442000, China

**Keywords:** duck Tembusu virus, protein, innate immune response, prevention and control, vaccine

## Abstract

Duck Tembusu Virus (DTMUV) is a pathogen of the Flaviviridae family that causes infections in poultry, leading to significant economic losses in the duck farming industry in recent years. Ducks infected with this virus exhibit clinical symptoms such as decreased egg production and neurological disorders, along with serious consequences such as ovarian hemorrhage, organ enlargement, and necrosis. Variations in morbidity and mortality rates exist across different age groups of ducks. It is worth noting that DTMUV is not limited to ducks alone; it can also spread to other poultry such as chickens and geese, and antibodies related to DTMUV have even been found in duck farm workers, suggesting a potential risk of zoonotic transmission. This article provides a detailed overview of DTMUV research, delving into its genomic characteristics, vaccines, and the interplay with host immune responses. These in-depth research findings contribute to a more comprehensive understanding of the virus’s transmission mechanism and pathogenic process, offering crucial scientific support for epidemic prevention and control.

## 1. Introduction

The Duck Tembusu Virus (DTMUV) belongs to the Flaviviridae family, Flavivirus genus, and the Ntaya virus group [1]. To date, there are over fifty viruses in the Flavivirus genus, including the Japanese encephalitis virus, Dengue virus, and Zika virus, which can cause severe diseases in both humans and animals [2,3]. Duck Tembusu Virus disease is an acute infectious disease caused by DTMUV infection in ducks, leading to significant economic losses in the duck farming industry in a short period, posing a serious threat to its development [4,5]. The main clinical symptoms of affected ducks include a rapid decline in egg production, ataxia, severe ovarian hemorrhage, ovarian inflammation, splenomegaly, hepatomegaly, splenic necrosis, and brain necrosis [6,7]. The morbidity in adult ducks ranges from 10% to 30%, while in ducklings, it can reach 90% to 100%, with a mortality rate of 5% to 30% [8]. Some studies have shown that DTMUV has a broad host range and various transmission routes, including vertical transmission [9] and mosquito-borne transmission [10], as well as direct contact and airborne transmission [11,12]. Besides infecting ducks, it can also infect chickens, geese, pigeons, and sparrows [13,14,15]. Literature reports have detected neutralizing antibodies against DTMUV in the serum of duck farm workers, although it has not been demonstrated to be pathogenic to humans, indicating potential zoonotic transmission between ducks and humans. This should arouse our high attention and prompt action [5,9,16].

**Figure 1:**
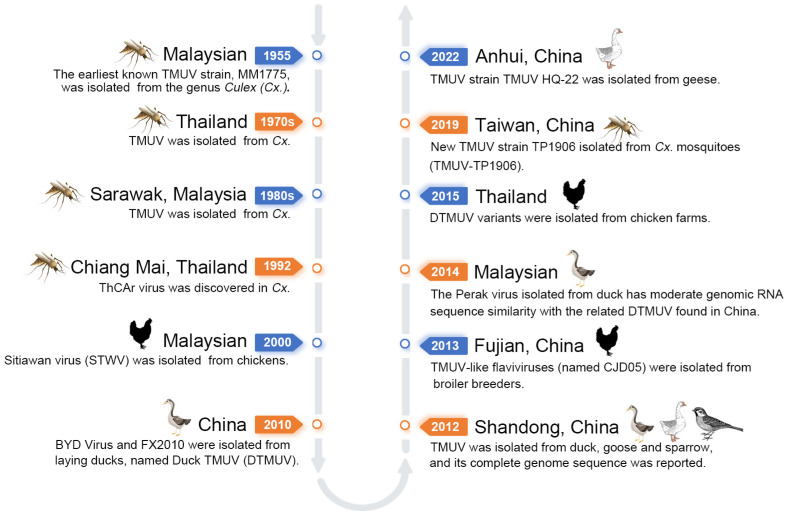
Timeline of the history of TMUV. The timeline illustrates the spread history of TMUV, the infected hosts, and typical strains isolated since 1955.

The earliest known strain of TMUV, MM1775, was isolated in 1955 from mosquitoes of the genus *Culex* (*Cx.*) *triteaniorhynchus*, *Cx. gelidus*, *Aedes linneatopennis*, and *Anopheles philippinensis* in the Malaysian Peninsula [17]. In the 1970s and 1980s, TMUV was successively isolated in Thailand from *Cx. gelidus*, *Cx. tritaeniorhynchus*, *Cx. Vishnui*, and *Cx. Sitiens*, and in Sarawak, Malaysia, from *Cx. gelidus*, *Cx. pseudovishnui*, and *Cx. tritaeniorhynchus* [18]. In 1992, the ThCAr virus was discovered in *Cx. tritaeniorhynchus* mosquitoes captured in Chiang Mai, Thailand, with no observed disease association [19]. In the following decades, sporadic cases of TMUV isolation were reported only in Southeast Asian countries [17]. In 2000, an outbreak occurred in a broiler chick farm in Malaysia, causing an infectious disease characterized by ataxia and delayed growth in chicks aged 4–6 weeks. A flavivirus was found in affected chickens and named Sitiawan virus (STWV) [20]. In April 2010, severe duck virus infections broke out in several duck farms in southeastern China, and a new virus named BYD Virus was isolated from ducks suffering from duck egg drop syndrome [21]. In the same year, during an investigation into the epidemic of decreased egg production, slow growth, and even death in ducks in southeastern China, a novel strain of TMUV, named Fengxian 2010 (FX2010), was isolated. The partial E protein of the TMUV MM1775 strain neutralized FX2010, with a nucleotide sequence identity of 99.6% to BYD Virus and 88% to TMUV. It was named DTMUV [22]. In 2012, duck isolates (YY5 and ZJ-407) and goose isolates (GH-2) were isolated from ducks, breeder ducks, and geese in Shaoxing, China, and their complete genome sequences were reported [23]. In 2012, TMUV strain TMUV-SDHS was isolated from sparrows around poultry farms in Shandong Province, northern China [14]. In 2013, TMUV-like flaviviruses (named CJD05) were isolated from broiler breeders with severe egg drop and fever in Fujian Province, China [24]. In 2012, a Malaysian DTMUV strain named Perak Virus was isolated from several duck farms in Malaysia, showing moderate similarity in genomic RNA sequences to DTMUV found in China [25]. In 2015, DTMUV variant strains were isolated from layer and broiler chicken farms in Thailand [26]. In 2019, a new strain of TMUV, TMUV-TP1906, was identified and isolated from mosquito pools collected in northern Taiwan. Its similarities to STWV and TMUV prototype strain MM1775 were 93.71% and 91.27%, respectively, marking the first isolation of TMUV from mosquitoes [27]. In 2022, the TMUV strain TMUV HQ-22 was isolated from infected geese in Anhui, China. Compared to other typical TMUV strains, it exhibited mutations in the envelope (E) protein and showed significant pathogenicity in goslings and ICR mice [15] (Figure 1).

In recent years, researchers have conducted extensive studies on DTMUV, making progress in understanding various aspects from the virus’s genome structure to its pathogenic mechanisms, transmission routes, and host immune responses [11,28]. The analysis of the virus’s genome characteristics and protein structures has helped deepen our understanding of its lifecycle and pathogenic mechanisms, providing important clues for vaccine development and drug therapy. Although vaccines and detection methods have been developed, there are still many unresolved issues that require further research, such as the virus’s long-term transmission dynamics and host immune mechanisms against the virus [29]. Vaccine development not only provides preventative protection to alleviate the impact of the disease on poultry farming but also helps to curb the spread of the virus. However, the role of innate immune responses as the body’s first line of defense against DTMUV infection is also of great interest.

This paper, through reviewing epidemiology, genomic characteristics, vaccines, and the interplay with host immune responses, contributes to a better understanding of the virus’s infection mechanisms. It provides important scientific support for epidemic prevention and control, offers a comprehensive perspective for understanding and addressing DTMUV control strategies, and serves as a valuable reference for research and practices in related fields.

## 2. Genomic Characteristics

**Figure 2:**
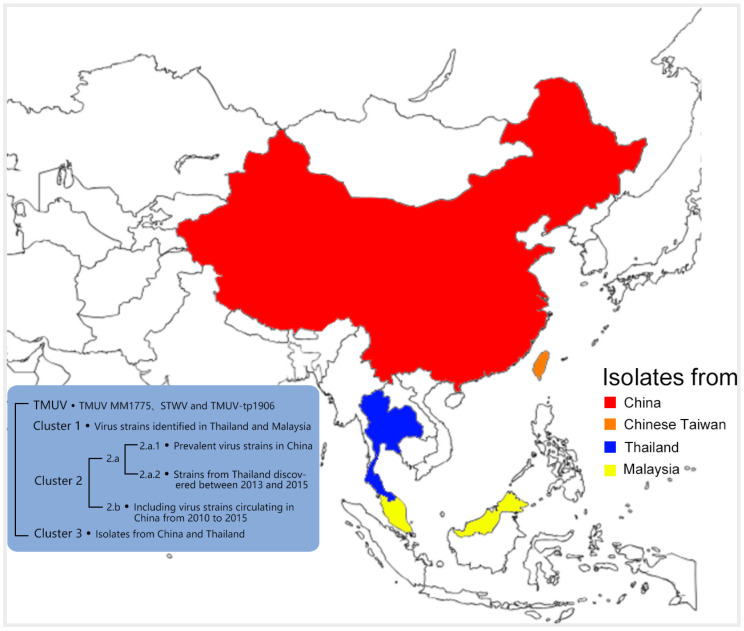
Model diagram of TMUV distribution and typing. TMUV strains have primarily been isolated in Thailand, Malaysia, and China. They are categorized into four main clusters: TMUV, Cluster 1, Cluster 2, and Cluster 3.

Since the early 21st century, there have been successive reports of TMUV circulation in East Asia and Southeast Asia. However, during the continuous circulation, these virus isolates have undergone cluster separation and diverged phylogenetically from the TMUV cluster. Based on the reported full-length genome sequences of TMUV and the ML trees of the E, NS1, and NS5 gene sequences, they are divided into four major phylogenetic clusters: TMUV, Cluster 1, Cluster 2, and Cluster 3. The TMUV cluster contains the first TMUV virus isolated in 1955 (TMUV MM1775), strains discovered in Sitiaowan in 2000 (STWV), and strains found in Taiwan, China in 2019 (TMUV-TP1906). The virus strains isolated in Thailand in 2007 and two strains isolated in Malaysia in 2012 form a monophyletic lineage, named Cluster 1. Over the past decade, most strains isolated in China and Thailand have formed a branch called Cluster 2, which is further subdivided into two subgroups: Cluster 2.a and Cluster 2.b. Since 2010, most strains isolated in Thailand and a small number from China belong to Cluster 2.a, while most isolates from China are assigned to Cluster 2.b. In recent years, two different research groups have identified a new cluster called Cluster 3 within the TMUV group, which collects virus isolates from China and Thailand [15,30,31,32,33] (Figure 2).

**Figure 3:**
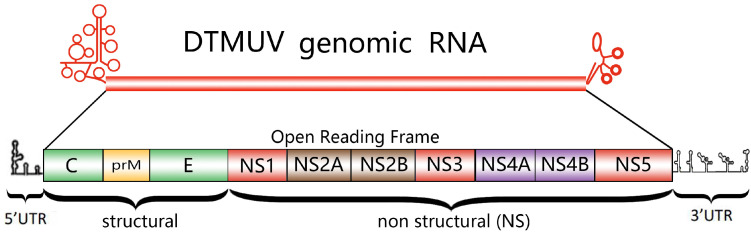
The DTMUV genome. The DTMUV genome contains a single major open reading frame flanked by untranslated regions. The 10 genes within the open reading frame are indicated.

The genome of DTMUV consists of a non-segmented, single-stranded positive-sense RNA, approximately 11 kb in length [34]. Mature DTMUV particles are spherical, with a diameter of 50 to 75 nm, exhibiting a typical enveloped flavivirus structure. The RNA core appears dark and dense, with the capsid and lipid bilayer clearly visible. The capsid forms regular hexagons, and the outer structural domain of the E glycoprotein appears striped rather than showing visible spikes [35]. The genome comprises a single open reading frame (ORF), flanked by 5′ and 3′ untranslated regions (UTRs). The 5′ end possesses an m7GppAmp structure (cap 1), while the 3′ end lacks a poly-A tail [36]. This ORF encodes a polyprotein precursor containing 3425 amino acids. Subsequently, the polyprotein undergoes cleavage by the host signal peptidase, furin protease [37], and the virus’s serine protease NS2B/NS3. This results in the formation of three structural proteins (capsid protein, prM membrane protein, and envelope protein E) involved in the assembly of infectious viral particles and the virus’s cellular entry and release processes. Additionally, seven non-structural proteins (NS1, NS2A, NS2B, NS3, NS4A, NS4B, and NS5) play roles in viral transcription and replication, concurrently participating in the modulation of the host’s antiviral innate immune response [38,39] (Figure 3).

### 2.1. Structural Proteins

DTMUV capsid protein (C) has a molecular weight of approximately 13 kDa. It is an asymmetrically charged dimeric protein, with a highly cationic region responsible for binding to viral RNA and forming the virus ribonucleoprotein, while its hydrophobic region interacts with the envelope glycoprotein E. The flavivirus capsid protein not only forms the viral capsid to protect the viral RNA but also interacts with various host proteins to facilitate viral replication. Therefore, the capsid protein plays a crucial role in infected host cells and the viral lifecycle [40,41]. Researchers utilized reverse genetics techniques, using TMUV as a model, to express the mature capsid protein (CP) under the control of either the endogenous ribosomal entry site (IRES) or the minimal IRES in the 3′ untranslated region (3′UTR). The original capsid gene was deleted. Recombinant viruses exhibited a significantly attenuated phenotype and severely reduced proliferation in vitro [42].

The DTMUV premembrane protein (prM) has a molecular weight of approximately 18 kDa. When it is cleaved by the furin protease located in the trans-Golgi network into its M form, the viral particles mature. The M form consists of 75 amino acid residues, with a protein molecular weight of approximately 8 kDa [43,44]. PrM plays a crucial role in promoting the secretion of the E protein. Additionally, prM plays a vital role in the folding, stability, and protective immune development of the E protein [44,45,46].

The DTMUV envelope protein E has a molecular weight of approximately 53 kDa. It is the main surface protein of the viral particles, mediating the binding of the virus to cellular receptors and subsequent fusion with the host membrane [47]. Similar to other flavivirus E proteins, the DTMUV E protein is an important target for neutralizing antibodies [48]. Expression of the DTMUV E protein in Bacillus subtilis induces immune protection in ducklings [49]. Likewise, expression of the DTMUV E protein in a pseudorabies virus induces protective immunity in ducklings [50]. The E protein of flaviviruses exists on the viral membrane as homodimers, consisting of three domains (the ectodomain, stem region, and transmembrane domain), each with distinct functions, forming the basis for viral replication and virulence [47,51,52]. The D120N amino acid mutation located in domain II of the E protein is a critical molecular basis for the high attenuation and loss of transmission capability of the TMUV vaccine FX2010-180P [53]. Furthermore, studies on functional antibodies and epitope mapping indicate that all three domains are antigenic, capable of being recognized by neutralizing antibodies and inhibiting the virus entry process, such as viral binding to host cell surfaces and fusion with host membranes [54,55,56].

### 2.2. Non-Structural Proteins

DTMUV NS1 has a molecular weight of approximately 40 to 50 kDa. It is a highly conserved glycoprotein that exists on the cell surface or in association with cell-related proteins [57]. Despite the absence of a transmembrane domain, NS1 forms hydrophobic dimers on the cell membrane once it reaches the cell surface [58]. NS1 is one of the few NS genes capable of conducting retrograde complementation in stable expression replicase complexes [59]. NS1 plays a pivotal role in viral RNA replication, antiviral immunity, and host recognition of virus-associated molecular patterns [60,61].

DTMUV NS2A and NS2B are crucial in constructing the replicase complex and play pivotal roles in virus replication. NS2A has a molecular weight of approximately 25 kDa and is a small transmembrane protein containing eight transmembrane domains located on the endoplasmic reticulum, contributing to endoplasmic reticulum reorganization [62]. NS2A interacts with the 3′ UTR of viral RNA and other components of the replicase complex, indicating its involvement in viral RNA replication [63]. Additionally, the flavivirus NS2A protein is implicated in regulating host antiviral interferon responses [64]. Apart from being a component of the replicase complex, some mutational studies suggest that flavivirus NS2A participates in virus particle assembly/secretion and virus-induced cytopathic effects [65,66,67]. NS2B has a molecular weight of about 14 kDa. It is a transmembrane protein whose connection with NS3 is autocatalytically cleaved by the viral protease, resulting in NS3 conformational changes, converting NS3 into the more active and stable NS2B/3 protease. This complex is responsible for cleaving various viral proteins [68,69]. Through comparison with yellow fever virus NS2B, conserved amino acids were site-directed mutagenized, revealing that the mutations P32A, G36A, and G37A completely blocked viral RNA synthesis. The mutations D25A, A43Y, V44A, G92A, and P112A led to minor defects in virus replication. Only the G92A mutation affected virus proliferation by blocking virus release, without affecting viral RNA synthesis, indicating the involvement of G92 in the assembly of infectious TMUV particles [70].

DTMUV NS3 has a molecular weight of approximately 68 kDa and contains an N-terminal domain with a serine protease (peptidase_S7), while its C-terminal region harbors NTPase and RNA helicase activities (designated as Flavi_DEAD and HELICc, respectively) [71]. The NS3 N-terminal domain peptidase_S7 associates with the cofactor NS2B to form the NS2B-NS3 complex. NS3′s C-terminus encompasses three distinct enzyme activities: RTPase, NTPase, and helicase [72]. RTPase cleaves the phosphate bond of 5′-phosphorylated RNA and is considered the first step in the three consecutive enzymatic reactions required for the formation of RNA 5′ caps, crucial for viral translation and RNA stability [73]. The RTPase cleaves the phosphodiester bonds of 5′-triphosphorylated RNA, typically considered the first step of the three consecutive enzyme-catalyzed reactions for RNA 5′-end capping, which is crucial for viral translation and RNA stability [74,75]. The hydrolysis of NTPases provides energy, facilitating the viral unwinding and translocation processes [76]. Current research suggests that the NS3 HELICc domain of flaviviruses can establish an ATP-dependent stable equilibrium between RNA unwinding and annealing, allowing the enzyme’s two opposing activities to be regulated by ATP concentration [51,77,78,79,80].

DTMUV NS4A has a molecular weight of around 14 kDa and is a membrane-associated protein featuring four transmembrane helices and a cytoplasmic N-terminal region. It associates with the viral replicase complex, inducing endoplasmic reticulum (ER) membrane rearrangements. Once the virus genome is translated within the ER, NS4A is cleaved by the NS2B-3 viral protease complex in the cytoplasm [81]. The transmembrane domain of NS4A is cleaved by ER luminal host signalases [82]. Mature NS4A promotes membrane rearrangements by inducing curvature of the ER membrane [83]. NS4A and NS1 must interact to effectively synthesize viral RNA, implying that NS4A serves as a docking site for the replicase complex [83,84]. NS4A also indirectly binds double-stranded RNA by associating with host cell polyubiquitin-binding proteins, stabilizing the intermediate dsRNA genome [85].

DTMUV NS4B has a molecular weight of approximately 28 kDa and is the largest hydrophobic NS protein, featuring five transmembrane segments in a similar membrane topology. It is vital for NS4B’s roles in viral replication and interactions with host proteins [86]. As a part of the replicase complex, NS4B plays a crucial role in replication and assembly. Recent studies report NS4B’s self-oligomerization and interactions with NS1, NS2B, NS3, and NS4A [87,88,89,90,91].

DTMUV NS5 has a molecular weight of approximately 100 kDa and is the largest and most conserved protein, serving as the core of viral replication. NS5 features an N-terminal methyltransferase (MTase) domain and a C-terminal RNA-dependent RNA polymerase (RdRp) domain [92]. MTase is crucial for cap formation (GpppA-RNAm→7GpppAm-RNA) of the viral RNA genome, protecting the capped 5′ RNA from extracellular exonucleases and enhancing ribosome recognition for translation [93]. RdRp is responsible for the de novo synthesis of positive-strand RNA without additional cellular factors, recognizing the 5′ untranslated region (UTR) and synthesizing the negative strand by long-range RNA-RNA interactions to initiate the 3′ UTR [94,95]. NS5 is considered a crucial component of virus propagation [96].

## 3. Diagnostic Methods

Detection methods for DTMUV include virus isolation, multiplex RT-PCR techniques, enzyme-linked immunosorbent assay (ELISA), immunochromatographic strip (ICS) assays, and nanoparticle-assisted PCR methods. While virus isolation and identification methods are classic and accurate, their longer turnaround times make them more suitable for experimental research rather than rapid clinical diagnostics. Ninvilai et al. developed a one-step reverse transcription PCR targeting the highly conserved region of DTMUV’s NS5 gene, offering high accuracy, specificity, and sensitivity for routine DTMUV detection and epidemiological monitoring. It can widely detect all DTMUV clusters and is capable of detecting DTMUV as low as 0.001 50% embryo lethal dose per milliliter [97].

Given the development of mixed farming systems, increased human and animal mobility, and environmental contamination concerns, duck viral infections have become increasingly serious in recent years, often involving mixed infections. Therefore, establishing a multiplex RT-PCR method is essential for the rapid diagnosis of common duck viral diseases and cost-effective testing. Dual qPCR based on TaqMan enables the rapid diagnosis of DTMUV and Goose Astrovirus Genotype 2 (GoAstV-2), with detection limits of 100 copies/μL and 10 copies/μL, respectively [98]. Wang et al. established a multiplex qPCR capable of simultaneously detecting six duck-origin viruses, including DTMUV. The multiplex qPCR system can amplify six DNA fragments from the combined viral genome when the template concentration is 10^2^ copies/μL, and it can specifically detect the nucleic acids of six duck-sensitive viruses, improving efficiency, reagent conservation, and cost savings [99]. Yin et al. developed a multiplex qPCR assay capable of simultaneously detecting Duck Circovirus (DuCV), DTMUV, Muscovy Duck Reovirus (MDRV), and Novel Duck Reovirus (NDRV). However, it does not amplify other viruses, including Duck Viral Enteritis Virus (DVE), Infectious Bursal Disease Virus (IBDV), Avian Rotavirus (ARV), H5 Avian Influenza Virus (H5 AIV), H7 Avian Influenza Virus (H7 AIV), H9 Avian Influenza Virus (H9 AIV), Newcastle Disease Virus (NDV), and Muscovy Duck Parvovirus (MDPV). The detection limits for DuCV, DTMUV, MDRV, and NDRV are 1.51 × 10^1^ copies/μL [100]. The developed multiplex digital PCR (dPCR), compared to multiplex qPCR, can specifically detect DTMUV, DuCV, and NDRV but cannot amplify MDRV, MDPV, Goose Parvovirus (GPV), H4 Avian Influenza Virus (H4 AIV), H6 Avian Influenza Virus (H6 AIV), or NDV. Multiplex dPCR exhibits high sensitivity, with a detection limit as low as 1.3 copies/μL, which is 10 times lower than that of multiplex qPCR [101].

In serological testing, ELISA is commonly used to detect DTMUV antigens and antibodies. An Epitope-ELISA serological diagnostic method was developed using the specific antigenic epitope YAEYI of DTMUV’s E protein. Antigens based on YAEYI epitopes exhibit highly specific reactivity with serum samples obtained from ducks infected with DTMUV, without any serological cross-reactivity with West Nile virus (WNV), Dengue virus (DENV), and Japanese encephalitis virus (JEV), and can be applied for clinical diagnosis and antibody level evaluation [102]. The ELISA based on purified NS1 protein demonstrates no cross-reactivity with common duck pathogens such as H5N1 avian influenza, NDV, Duck Hepatitis Virus Type 1 (DHV-1), Duck Reovirus (DRV), Duck Plague Virus (DPV), and Riemerella anatipestifer (Duck Septicemia Agent). This ELISA can be used for the large-scale diagnosis and serological monitoring of DTMUV infection in China [103]. Based on the closed ELISA of monoclonal antibodies (MAbs) against TMUV, the specificity and sensitivity were 96.37% and 100%, respectively, with a kappa value of 0.966. This provides a reliable and rapid diagnostic tool for the serological monitoring and evaluation of TMUV infection [104]. By validating and comparing the hemagglutination inhibition (HI) and indirect immunofluorescence (IFA) methods for detecting DTMUV antibodies in duck serum, it was found that both methods have high sensitivity (100%) and specificity (>87%) and show over 90% agreement with the serum neutralization (SN) test results. Compared to the IFA test, the HI method demonstrated higher specificity and a stronger correlation with the SN test [105].

Immunochromatographic strip (ICS) assays are widely employed in clinical diagnostics due to their rapidity, minimal equipment requirements, high specificity, and sensitivity. Researchers have developed a novel ICS detection method using the colloidal gold-labeled monoclonal antibody A12D3 targeting the envelope E protein of DTMUV. This ICS can specifically detect DTMUV within 10 min. By simultaneously testing 50 clinical samples using ICS and RT-PCR, the concordance rate between the two methods reached 93.9% [106]. A nano PCR detection method targeting the DTMUV E gene demonstrates a sensitivity ten times higher than traditional PCR detection. The detection limit of nano PCR is 1.8 × 10^2^ copies/μL of DTMUV RNA, with no cross-reactivity observed with other viruses [107,108]. Rational application of these methods in practical production facilitates rapid diagnosis, aiding in the development of appropriate prevention and control strategies.

## 4. Vaccination

In the absence of efficient and cost-effective treatment measures, vaccine immunization is one of the most effective measures for preventing and controlling DTMUV disease. Currently, commercially available vaccines mainly include inactivated DTMUV vaccines (HB strain, DF2 strain) and live DTMUV vaccines (WF100 strain, FX2010-180P strain) (Table 1). Meanwhile, gene recombinant vaccines, DNA vaccines, and subunit vaccines are also current research focuses.

The Tembusu-HB strain of DTMUV was converted into an inactivated vaccine using formaldehyde inactivation and mineral oil emulsification. After two intramuscular or subcutaneous injections of the inactivated vaccine, more than 80% of immunized ducks showed good immunogenicity and resistance to the virulent strain of DTMUV. It is interesting that varying levels of protection (20–80%) were observed in Beijing white geese when vaccinated twice with the same batch of vaccine. This suggests that the vaccine exhibits species-specific effects [109]. Another example is the β-propiolactone (BPL)-type inactivated DTMUV vaccine prepared by successfully using BPL inactivation and mineral oil emulsification on the TMUV-JXSP strain cultivated in Baby Hamster Kidney (BHK-21) cells. After the second immunization to enhance efficacy, the vaccine induced antibody responses in most ducks, providing high-level protection for laying ducks. Single-dose vaccine immunization resulted in a protection index of 87%, significantly reducing viral load in tissues and meat ducks against virus attacks [110]. Additionally, the DTMUV AH-F10 strain oil emulsion inactivated vaccine, when subcutaneously administered to 120-day-old laying ducks, led to a gradual recovery in total egg production starting from the 4th day after immunization, returning to pre-immunization levels by the 20th day, demonstrating certain safety and effective stimulation of ducklings to produce immune antibodies [111]. Furthermore, the DTMUV YZDTV-18 strain inactivated vaccine, prepared by formaldehyde inactivation and formulated into an oil emulsion, showed no significant differences in feed consumption or egg production rate, and no typical clinical symptoms or pathological changes in laying ducks after challenge at 45 weeks of age, indicating a certain level of immune protection for laying ducks [49]. Combining inactivated DTMUV vaccines with cytidine–phosphate–guanine oligodeoxynucleotides (CpG ODNs) and interleukin-2 (IL-2) can increase antibody levels and cytokine levels, enhancing the immune-protective effects of the vaccine. Therefore, pUC18-CpG and IL-2 can serve as adjuvants for inactivated DTMUV vaccines [112,113].

Currently, commercially available live attenuated DTMUV vaccines in China include the DTMUV live vaccine FX2010-180P and the DTMUV live vaccine using the WF100 strain. FX2010-180P utilizes the FX2010 strain, passaged continuously on chicken fibroblast cells, and the purified strain obtained is non-pathogenic in ducks aged 3 to 5 weeks but can induce a strong immune response and protect ducks from the FX2010 strain. During passaging, 19 amino acid missense mutations and 15 amino acid synonymous mutations occurred, including mutations in multiple proteins such as prM/M, E, NS1, NS3, NS4A, NS4B, and NS5. This vaccine cannot spread infection through the nasal route and horizontal transmission to susceptible ducks. When administered with a low dose via intramuscular injection, it induces a good immune response, and the duration of immunity is over 6 months [114]. Another example is the SDS-70 strain, developed from the DTMUV SDS strain isolated from sparrows, passaged continuously in specific-pathogen-free duck embryos, and artificially attenuated to form the SDS-70 strain. SDS-70 provides certain immune protection against the virulent SDS-101 strain [115]. DTMUV with MTase deficiency exhibits reduced virulence and induces higher innate immunity, providing protection to ducks against lethal doses of DTMUV-CQW1 [116].

Truncated DTMUV E protein expressed using a rod-shaped virus expression system can induce specific antibodies in ducklings, making it a potential candidate vaccine for preventing duckling infections [117]. Vaccination of ducklings with recombinant rod-shaped viruses containing the DTMUV E gene induces strong humoral and cellular immune responses, providing 100% protective immunity against the DTMUV YY5 strain [50]. Additionally, the fusion of the E protein with the epitope protein rTBE has immunogenicity and protective efficacy, making it a potential safe and effective novel DTMUV subunit vaccine [118]. Recombinant carrier vaccines developed using adenovirus and Salmonella have been shown to stimulate a higher level of cellular immune response and produce high levels of neutralizing antibodies, providing 100% protective efficacy against challenge [119]. A recombinant Semliki Forest virus (SFV) replicon highly expressing the E protein in duck embryo fibroblast (DEF) cells, when used as a DNA vaccine through muscle injection in ducklings, induces strong humoral and cellular immune responses and resistance against the virulent AH-F10 strain [120].

The amino acid sequence of TMUV NS1 exhibits high conservation across different strains (92.63–100%). Plasmid immunization platforms constructed based on TMUV NS1 can stimulate specific anti-NS1 IgG responses without inducing neutralizing antibodies against TMUV. NS1 demonstrates significant protective effects against TMUV attack without causing severe gross lesions [60]. Moreover, oral immunization of ducklings with Bacillus subtilis secreting DTMUV E protein effectively enhances resistance to DTMUV and significantly reduces virus titers (*p* < 0.01) while reducing pathological damage to the brain, heart, and spleen [49]. Furthermore, self-assembled nanoparticles carrying the structural domains I, II, and III of DTMUV E protein (EDI-II-RFNp, EDIII-RFNp) prepared using ferritin as a carrier can effectively protect ducks from DTMUV infection [121,122].

Through the analysis of the full genome sequence of various DTMUV strains, it has been found that DTMUV exhibits high genetic diversity. Whether current inactivated and live attenuated vaccines provide immune protection against all strains is still unknown and requires further research [11,15].

## 5. Innate Immune Responses

Numerous studies have investigated the innate immune response to DTMUV in avian and mammalian hosts both in vivo and in vitro. Female ducks and ducklings were experimentally infected with DTMUV, and it was found that the virus replicated rapidly in various organs (brain, spleen, kidney, heart, pancreas, thymus, and bursa of Fabricius). Infection led to the activation of the RLR and TLR signaling pathways and overexpression of PRRs such as RIG-I and MDA-5. This activation resulted in the upregulation of interferon-stimulated genes (ISGs) from the Mx and OAS families in the brain and spleen [123]. Injection of 10^4^ TCID50 of TMUV-TC2B into 5-day-old ducklings via a vein resulted in a significant upregulation of ISGs in the immune organs, causing dynamic damage to the immune system and providing insights into pathogenicity [124]. On the first day after DTMUV infection, the spleen exhibited the highest virus titers, with upregulation of Rig-1, Mda5, and Tlr3 expression, as well as the upregulation of pro-inflammatory cytokines (such as Il-1β, Il-2, Il-6, and Cxcl8) and antiviral proteins (such as Mx and Oas), with Il-6 showing the most significant increase in expression across all tested tissues [125]. In ducks infected with DTMUV, there was a significant negative correlation between the levels of CD4+ and CD8+ T, B, and non-T and -B lymphocytes and the viral load in the blood and target organs (spleen). Additionally, significant neutralizing antibody responses were observed [126]. DTMUV infection upregulates suppressor of cytokine signaling 1 (SOCS1), leading to ubiquitination and proteasomal degradation of IRF7, ultimately inhibiting the production of type I IFNs and promoting viral replication [127].

Hua et al. elucidated the importance of TBK1 in an experimental duck model, as TBK1 is involved in multiple type I IFN signaling pathways. Through overexpression and knockdown experiments, they proposed a crucial role for TBK1 in mediating IFN-β production in the antiviral innate immune response in DEF [128]. Avian IRF1 and IRF7 may regulate the expression of IFN-β and VIPERIN, as well as many other ISGs, to inhibit the replication of DTMUV through IFN-dependent and/or -independent mechanisms [129]. DEF cell-derived exosomal miR-148a-5p promotes DTMUV replication by negatively regulating TLR3 expression [130]. In ducks infected with DTMUV, the duck-DDX3 (duDDX3) protein modulates the innate immune response, and overexpression of duDDX3 suppresses TMUV. However, despite the potential impact of duDDX3 on DTMUV replication, the virus can inhibit the expression of duDDX3, suggesting the existence of potential mechanisms for immune evasion [131]. A series of experiments involving infection of CEF and 293T cells also demonstrated the significance of RLR and TLR pathways in the innate immune response to DTMUV infection. Infection with DTMUV strongly increased the expression of a set of type I IFN genes and key ISGs (Mx1, OAS1, IFITM3, and OASL) through activation of the molecular adaptors MDA5 and TLR-3 involved in the RLR and TLR pathways, respectively, leading to decreased viral replication [132]. Based on Stable Isotope Labeling by Amino Acids in Cell Culture (SILAC), protein profiling analysis of duck embryo fibroblast (DEF) cells infected with DTMUV revealed an upregulation of duck interferon-induced protein 35 (duIFI35). Moreover, duIFI35 was found to inhibit the activation of the IFN-β promoter induced by duck retinoic acid-inducible gene I (duRIG-I), thereby disrupting the duRIG-I-mediated host antiviral response and aiding DTMUV evasion of the host innate immune response [133].

The aforementioned studies highlight that DTMUV infection can elicit a robust immune response, but the virus must first overcome the host’s innate antiviral immune barriers to establish successful infection [134]. To replicate effectively within the host, flaviviruses must develop complex strategies to evade or subvert the host’s innate immune response. DTMUV has developed several strategies to disrupt innate immunity and promote productive infection. For instance, DTMUV can encode various NS proteins that inhibit the production of IFN-β by targeting similar cellular components [135].

Research indicates that the non-structural protein NS1 of DTMUV interacts with IPS1 through RIG-I and MDA5, impairing the RLR receptor signaling cascade. NS1 protein interacts with the CARD domains of RLR adapters, hindering the recognition and association with viral moieties. Disruption of this interaction inhibits RLR-mediated IFN-β production, ultimately promoting immune evasion. DTMUV NS1 disrupts the RLR pathway in HEK293 cells by targeting STING, thereby inhibiting virus-induced IFN-β expression [136].

DTMUV NS2A inhibits the IFN-β signaling pathway by competitively binding to STING, thereby reducing the phosphorylation of TBK1. The region spanning amino acids 114–143 of NS2A is crucial for its interaction with STING and inhibition of STING-mediated IFN-β signaling [137]. DTMUV NS2A competes with duTBK1 for binding to duSTING, disrupting duSTING-duSTING interaction and reducing duTBK1 phosphorylation, subsequently suppressing IFN-β production and compromising duSTING-dependent antiviral cellular defenses. Key residues in duSTING, including W164, Y167, and S361, are essential for this interaction [138].

The upregulation of the RLR genes RIG-I and MDA-5 early in the course of in vivo infection induced increased expression of type I IFNs [123,124,125].

Wu et al. investigated the role of the NS2B protein during viral infection of HEK293T cells and found its interaction with participants in the RIG-I pathway, leading to the inhibition of type I interferon responses. The NS2B-NS3 protease complex of the virus may also suppress the production of IFN-β. Unlike NS1, the NS2B-NS3 protease complex directly interacts with the duck STING protein in the mitochondria, a key intermediate in the RLR pathway, to inhibit signaling transduction and reduce IFN-β production. In this case, the synthesis rate of ISG-related proteins (Mx1 and OASL) is also downregulated [138,139]. Interestingly, the NS2B-NS3 proteinase complexes of Zika virus (ZIKV) and DENV have the same cleavage site in duSTING as DTMUV, suggesting similarities between DTMUV and other flaviviruses in their evasion mechanisms [140]. The NS2A protein’s role in RLR signaling transduction was also studied by the same research group, yielding similar results with changes in the activity of the STING protein [141]. Similar to NS2B, the binding of NS2A to STING is also associated with the inhibition of TBK1 phosphorylation, leading to the suppression of IFN-β production [138]. TMUV NS2B specifically interacts with mitochondrial antiviral signaling protein (duMAVS) to mediate ubiquitination and proteasomal degradation, thereby inhibiting the production of IFN [142]. NS2B is capable of inhibiting MDA5- or poly (I:C)-mediated IFN-β production, and this inhibitory effect is weakened when amino acid residues 51 to 92 (hydrophilic region) are deleted, indicating the importance of the hydrophilic region of NS2B for its interaction with the host innate immune system [143].

The DTMUV NS4B protein can inhibit IFN-β production. Among NS2A, NS2B, and NS4B, DTMUV’s NS4B protein has been identified as a key inhibitor of the RLR pathway, leading to decreased expression of RIG-I, MDA5, MAVS, STING, and TBK1. Moreover, specific mutations in the NS4B protein modify its interaction with TBK1, resulting in phenotypic changes and reduced pathogenicity of DTMUV. Thus, NS4B seems to strongly interact with TBK1 and inhibit its recruitment via STING, ultimately leading to pathway blockade [144]. These studies confirm the importance of TBK1 in executing effective antiviral responses, as reported by Hua et al., and reveal strategies employed by DTMUV to overcome immune responses [128].

The E218A mutation in the MTase domain of the DTMUV NS5 protein impairs virus replication and translation and activates RIG-I-like receptor signaling, ultimately leading to reduced virus proliferation [145]. DTMUV infection induces increased expression of duck TRAF3 (duTRAF3), which inhibits DTMUV replication. During this process, DTMUV NS5 interacts with TRAF3 and suppresses TRAF3 expression to counteract the transcriptional activity of IFN-α and its downstream ISGs [96].

**Figure 4:**
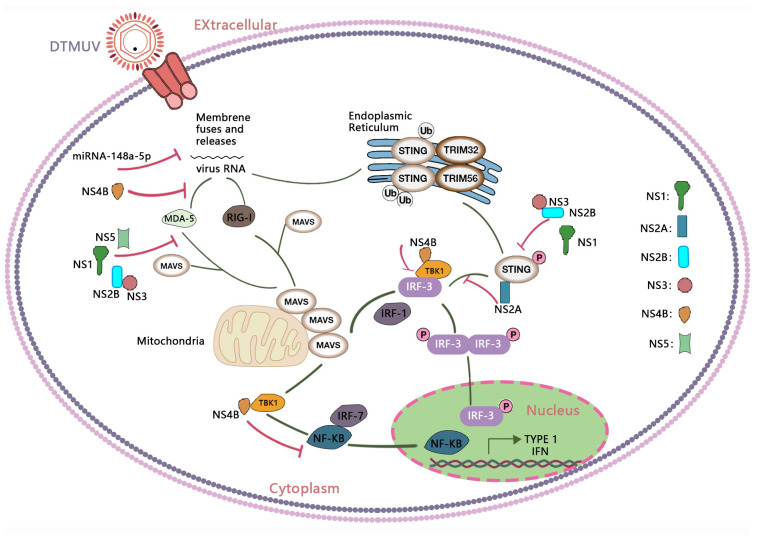
Immune response induced by DTMUV infection and its immune evasion. RIG-I-like receptors (RLRs) such as RIG-I and MDA5 can recognize the DTMUV genomic RNA and trigger signaling cascades through the adaptor protein MAVS, inducing the production of interferons (IFNs). DTMUV RNA activates the STING pathway and induces TBK1-IRF-3-mediated IFN expression. The IFN-mediated Janus-STAT signaling pathway induces abundant expression of various ISGs, including Mx1, OAS1, IFITM3, and OASL, which can inhibit DTMUV replication. To date, DTMUV has evolved multiple mechanisms to evade host immune responses, involving interactions between various viral proteins and host immune signaling pathways, including NS1, NS2A, NS2B, NS3, NS4B, and NS5.

Through these studies, we have gained a deeper understanding of the immune response during DTMUV infection. DTMUV triggers a series of immune reactions, including effects on immune organs, release of cytokines, and modulation of lymphocyte levels. However, DTMUV has also evolved multiple evasion mechanisms to suppress or disrupt the host’s innate immune response, thereby successfully establishing infection and promoting virus replication. These evasion strategies involve interactions between multiple viral proteins and host immune signaling pathways, including NS1, NS2A, NS2B, NS3, NS4B, and NS5, among others. Particularly noteworthy is the discovery of specific interactions between viral proteins and host immune molecules, such as the interaction between NS4B and TBK1. These findings provide important clues for a deeper understanding of the immunoregulatory mechanisms during DTMUV infection and offer valuable insights for future antiviral research and vaccine design (Figure 4).

## 6. Conclusions

Since the first outbreak of DTMUV infection in 2010, the duck farming industry has suffered immense economic losses, and the threat of this virus continues to persist. In recent years, researchers have made some progress in understanding the transmission modes, biological characteristics, epidemiological patterns, and development of DTMUV vaccines [146]. Vaccine development is one of the main strategies for DTMUV prevention and control. Various types of vaccines, including attenuated live vaccines, inactivated vaccines, and subunit vaccines, have demonstrated varying degrees of protective effects against DTMUV infection. The research on these vaccines provides diversified options for formulating comprehensive vaccination programs, ensuring more reliable protection for the poultry farming industry. While some DTMUV detection methods and vaccine formulations have been successfully launched in the domestic market, effectively curbing the spread and prevalence of certain strains, it is important to note that DTMUV belongs to the class of RNA viruses. RNA viruses have the propensity to mutate within the host environment, potentially leading to the ineffectiveness of existing detection methods and vaccines [11,15,30,147]. Additionally, with diverse transmission routes and a wide range of hosts, there is a potential risk of zoonotic transmission [11,15]. Therefore, there is still a huge demand in the duck farming industry for more effective and economically feasible vaccines. Thus, the development of safe and efficient DTMUV detection methods and vaccine formulations still requires further exploration.

Structural proteins C and E, as well as non-structural proteins NS1, NS3, and NS2A of DTMUV, are considered key targets for the specific detection of DTMUV or the development of nucleic acid vaccines. Utilizing these targets to the fullest extent helps accurately identify DTMUV and improve detection accuracy, thereby promoting its prevention and control. Although there are currently no specific drugs for treating DTMUV, besides strengthening farming management, proactive monitoring of DTMUV infections in duck populations in farming areas is also crucial for more effective prevention and control of its spread.

Research on DTMUV immune escape mainly focuses on non-structural proteins. However, structural proteins of other members of the Flaviviridae family also play important roles in virus infection. The specific roles of DTMUV structural proteins in virus infection and immune escape processes require further investigation. Moreover, despite active research on the molecular mechanisms, the patterns of host responses to infection are still not fully clear and require further study to elucidate the interactions between hosts and pathogens. The innate immune response plays a crucial role in DTMUV infection. The host’s innate immune system exhibits resistance to DTMUV by recognizing viral particles, activating inflammatory responses, and producing antiviral cytokines. Understanding the mechanisms of innate immune responses, especially the role of TLRs and other pathways, is expected to provide a basis for designing more targeted vaccines.

To effectively prevent and control the spread of DTMUV in the future, multifaceted measures need to be implemented. Firstly, strengthening epidemiological research to gain a deeper understanding of DTMUV transmission mechanisms, host range, and ecological characteristics is crucial for identifying key transmission routes and risk factors. Secondly, expanding surveillance efforts to cover not only duck farmers and farms but also surrounding communities and broader regions is essential for timely detection and response to outbreaks. Simultaneously, conducting public health education and awareness campaigns to increase awareness of DTMUV infection among high-risk populations and teach them effective personal protective measures is imperative. Additionally, developing vaccines targeting DTMUV to provide a scientific basis for preventing viral infections is essential. Establishing clear disease management guidelines to ensure prompt diagnosis and treatment for infected individuals while implementing isolation measures to reduce virus transmission is vital. Implementing biosecurity measures on farms to minimize the risk of virus transmission from animals to humans is critical. Implementing mosquito control strategies, as mosquitoes may serve as vectors for DTMUV transmission, is also important. Finally, maintaining data sharing and transparency to enable global health organizations and research institutions to promptly understand epidemic dynamics and assist in formulating effective response strategies is crucial. Through these comprehensive measures, the potential threat of DTMUV to human health can be minimized.

## Figures and Tables

**Table 1:** Duck Tembusu virus disease vaccines approved for use in China.

Product Name	Approval Time	Research and Development Unit
Inactivated vaccine of Duck Tembusu virus disease (HB strain)	5 May 2016	Beijing Academy of Agriculture and Forestry Sciences (Beijing, China), Reipu (Baoding) Biopharmaceutical Co., Ltd. (Baoding, Hebei, China), Yangzhou Unibio Pharmaceutical Co., Ltd. (Yangzhou, Jiangsu, China), and so on.
Live vaccine of Duck Tembusu virus disease (WF100 strain)	27 June 2016	Qilu Animal Health Products Co., Ltd. (Jinan, Shandong, China).
Live vaccine of Duck Tembusu virus disease (FX2010-180P strain)	15 October 2018	Shanghai Veterinary Research Institute, Chinese Academy of Agricultural Sciences (Shanghai, China), Jilin ZhenyeBiologic Products Co., Ltd. (Jilin, China), Qingdao Yibang Bioengineering Co., Ltd. (Qingdao, Shandong, China), and so on.
Inactivated vaccine of Duck Tembusu virus disease (DF2 strain)	26September 2021	Huazhong Agricultural University (Wuhan, Hubei, China), Wuhan Kexin Bio-Engineering Co., Ltd. (Wuhan, Hubei, China).

## Data Availability

Not applicable.
